# Biomaterial physicochemical properties govern immune activation and bone regeneration: a titanium-focused design-oriented osteoimmunological framework

**DOI:** 10.3389/fimmu.2026.1856086

**Published:** 2026-05-20

**Authors:** Żaneta Anna Mierzejewska, Jan Borys, Łukasz Woźniak, Jérôme R. Lechien, Luigi Angelo Vaira, Kamila Łukaszuk, Bożena Antonowicz

**Affiliations:** 1 Institute of Biomedical Engineering, Faculty of Mechanical Department, Bialystok University of Technology, Bialystok, Poland; 2 Department of Maxillofacial and Plastic Surgery, Medical University of Bialystok, Bialystok, Poland; 3 Department of Dental Surgery, Medical University of Bialystok, Bialystok, Poland; 4 Department of Surgery, Université de Mons (UMONS) Research Institute for Health Sciences and Technology, University of Mons, Mons, Belgium; 5 Department of Otolaryngology-Head and Neck Surgery, Foch Hospital, Paris Saclay University, Suresnes, France; 6 Maxillofacial Surgery Operative Unit, Department of Medicine, Surgery and Pharmacy, University of Sassari, Sassari, Italy

**Keywords:** biomaterials, bone regeneration, cytokine signaling, immunomodulation, innate immunity, macrophage polarization, osteoimmunology

## Abstract

The host immune response is increasingly recognized as a critical determinant of implant performance and bone regeneration in craniofacial applications. In osteoimmunology, macrophages act as central regulators of the foreign body response by integrating material-derived cues with intracellular signaling pathways that control inflammation and tissue repair. In this context, biomaterials actively regulate the immune microenvironment. However, the integration of biomaterial physicochemical properties with immune signaling and regenerative outcomes remains incomplete. Here, a mechanistic and design-oriented perspective on osteoimmunological processes governing biomaterial–tissue interactions is provided, with a particular focus on macrophage polarization, cytokine signaling networks, and apoptosis pathways involved in bone remodeling. Special attention is given to titanium wear particles as key immunological stimuli that activate macrophages through NF-κB, MAPK, and STAT signaling pathways, as well as emerging mechanisms including inflammasome activation and immunometabolic reprogramming. A unified osteoimmunological framework is introduced that integrates biomaterial physicochemical properties with immune signaling pathways and regenerative outcomes. Within this framework, material-induced modulation of macrophage phenotypes and cytokine profiles is identified as a central design axis controlling the balance between inflammation and regeneration. Emerging immunomodulatory strategies are discussed, including surface nanoengineering, ion-releasing systems, bioactive coatings, and stimuli-responsive biomaterials enabling spatiotemporal control of immune responses. Key limitations, including the oversimplified classification of macrophage phenotypes and the limited translational relevance of *in vitro* models, are critically addressed. By integrating immunology with materials science, this review outlines design principles for next-generation immuno-instructive biomaterials. This perspective supports the rational design of immuno-instructive biomaterials with predictable regenerative outcomes.

## 1 Introduction

Craniofacial bone defects arise from traumatic injuries, congenital malformations, tumor resections, and degenerative diseases, representing a significant clinical challenge in reconstructive surgery. The management of these defects frequently relies on biomaterials that provide mechanical stabilization and structural support, including metallic fixation systems, biodegradable polymers, and bioactive ceramics (1–3). Traditionally, the development of such biomaterials has focused primarily on mechanical strength, corrosion resistance, and structural integrity.

However, biomaterials are increasingly recognized as active regulators of the local biological environment. In particular, the host immune response has emerged as a critical determinant of long-term implant performance and tissue regeneration (4). Upon implantation, biomaterials interact with host proteins, immune cells, and surrounding tissues, initiating the foreign body response. This response involves immune cell recruitment, inflammatory signaling, and tissue remodeling processes that ultimately govern implant integration (5). At the molecular level, biomaterials are recognized by the innate immune system through pattern recognition receptors (PRRs), including Toll-like receptors (TLRs) and NOD-like receptors (NLRs). Activation of these receptors initiates intracellular signaling cascades, such as nuclear factor kappa B (NF-κB) and inflammasome pathways, leading to the production of pro-inflammatory cytokines and the recruitment of additional immune cells (4–6).

In parallel, biomaterial surfaces rapidly adsorb host proteins, which can trigger activation of the complement system, further amplifying inflammatory responses and modulating immune cell behavior at the biomaterial–tissue interface. These early recognition events represent a critical upstream layer of immune activation that links material-derived cues with downstream osteoimmunological processes.

Among immune cells, macrophages play a central regulatory role in biomaterial-associated responses. These cells exhibit high phenotypic plasticity and can adopt distinct functional states depending on microenvironmental cues. The dynamic balance between pro-inflammatory and pro-regenerative macrophage phenotypes has been shown to critically influence angiogenesis, osteogenesis, and overall healing outcomes (6, 7). In the context of craniofacial biomaterials, macrophage-mediated processes are further modulated by material-specific factors, including surface properties and the release of degradation products.

Titanium-based fixation systems, widely regarded as the gold standard in craniofacial surgery, may generate wear debris through mechanical and electrochemical processes. Titanium particles released into peri-implant tissues can activate macrophages and stimulate inflammatory signaling pathways, including the production of tumor necrosis factor-α (TNF-α), which plays a key role in regulating bone metabolism and immune responses (8–10). Inflammatory microenvironments may additionally trigger apoptosis pathways involving caspase-3, contributing to cellular turnover and tissue remodeling. These interconnected processes highlight the importance of understanding the mechanistic links between biomaterial properties, immune activation, and regenerative outcomes.

A comprehensive integration of biomaterial physicochemical characteristics with immune signaling pathways and their effects on bone regeneration remains incomplete. Current studies often address these aspects separately, limiting the ability to establish unified design principles for next-generation biomaterials.

Particular emphasis is placed on macrophage polarization, cytokine signaling pathways, titanium wear debris, and apoptosis mechanisms involved in bone remodeling. In addition, emerging engineering strategies aimed at designing immunomodulatory biomaterials capable of directing host responses toward regenerative phenotypes are discussed. By integrating immune signaling processes with material design parameters, this review aims to support the development of advanced craniofacial implants with improved biological performance. Given its clinical relevance, titanium is used here as a representative model biomaterial to illustrate osteoimmunological mechanisms.

Despite significant progress in both biomaterials engineering and immunology, current research remains largely fragmented, with physicochemical properties of biomaterials, immune signaling pathways, and regenerative outcomes often studied in isolation. This separation limits the development of predictive design strategies for next-generation biomaterials. Moreover, widely used conceptual models, such as the M1/M2 macrophage classification, fail to capture the complexity and dynamic nature of immune responses observed *in vivo*. By linking physicochemical properties of biomaterials with macrophage-mediated signaling, cytokine networks, and downstream osteogenic responses, we aim to establish a design-oriented perspective that enables rational development of immuno-instructive biomaterials for craniofacial applications. This review provides a design-oriented framework linking biomaterial physicochemical properties with immune activation and regenerative outcomes. The following sections are structured to systematically connect biomaterial physicochemical properties with immune activation mechanisms and downstream regenerative outcomes.

### 1.1 Conceptual osteoimmunological design framework

To address the disconnect between biomaterial design parameters and biological outcomes, we propose a structured osteoimmunological framework that explicitly links physicochemical properties of biomaterials with immune activation mechanisms and regenerative outcomes. The framework can be described as a three-level causal cascade. Material-derived inputs, including surface topography, chemistry, stiffness, and ion release, define the initial physicochemical interface and regulate protein adsorption and integrin-mediated cell adhesion. These cues are subsequently interpreted through immune sensing mechanisms, where pattern recognition receptors activate intracellular signaling pathways such as NF-κB, MAPK, and inflammasome pathways. These pathways drive macrophage activation states, regulate cytokine production, and initiate immunometabolic reprogramming. Ultimately, these processes determine regenerative outcomes, including bone formation, fibrous encapsulation, or chronic inflammation.

Critically, this cascade represents a continuous and interdependent process in which material-derived cues initiate immune responses that propagate through signaling networks to determine regenerative outcomes.

**Figure 1:**
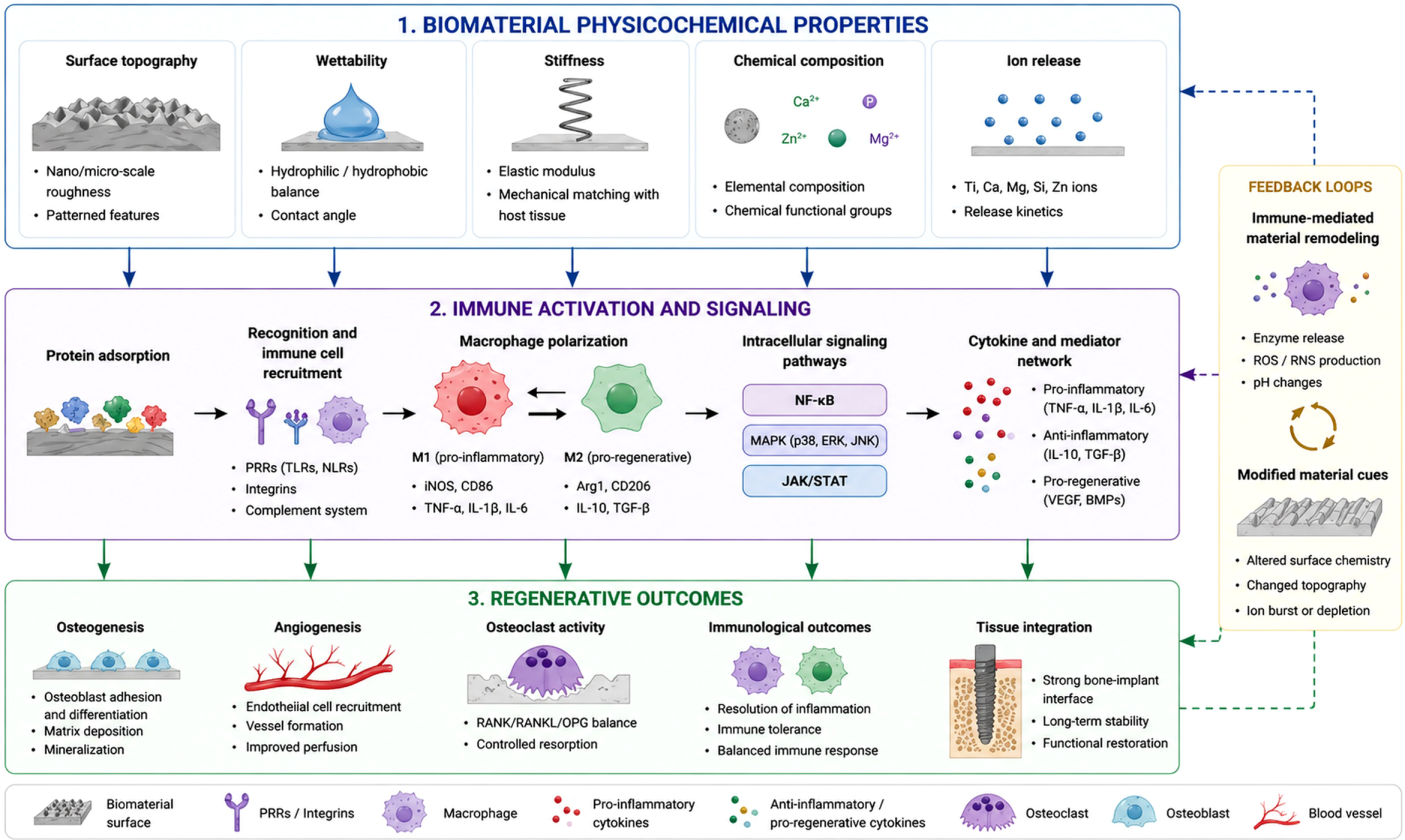
Design-oriented osteoimmunological framework linking biomaterial physicochemical properties with immune activation and bone regeneration. Biomaterial-derived physicochemical cues, including surface topography, wettability, stiffness, chemical composition, and ion release, regulate protein adsorption and immune cell interactions. These signals activate macrophage-mediated pathways and cytokine networks, which control osteogenesis, angiogenesis, and tissue remodeling. Feedback loops further modulate these interactions, highlighting immunomodulation as a key design variable (Created in BioRender (2026) https://BioRender.com/ggc2020).

**Figure 1** presents a design-oriented osteoimmunological framework integrating biomaterial physicochemical properties with immune activation and regenerative outcomes. Material-derived cues, including surface topography, wettability, stiffness, and chemical composition, regulate protein adsorption and immune cell interactions. These signals activate macrophage-mediated pathways and cytokine networks, which in turn control \osteogenesis, angiogenesis, and tissue remodeling. Feedback mechanisms further modulate these interactions, highlighting immunomodulation as a key design variable in biomaterials engineering.

These levels are dynamically interconnected through feedback mechanisms. For example, cytokine signaling can further modify cellular responses to biomaterial surfaces, while degradation products may continuously alter immune activation over time.

This framework establishes immunomodulation as a controllable design variable, enabling the rational development of biomaterials that actively guide immune responses toward regenerative outcomes. To translate this conceptual framework into actionable design principles, the relationships between material properties, immune activation pathways, and regenerative outcomes are systematically summarized in **Table 1**.

**Table 1:** Design-oriented osteoimmunological relationships.

Material parameter	Immune effect	Signaling pathway	Regenerative outcome
Surface roughness	Macrophage adhesion & polarization	NF-κB, FAK	Enhanced osteogenesis
Ion release (Ca^2+^, Mg^2+^)	Cytokine modulation	PI3K/Akt, STAT6	Pro-regenerative microenvironment
High stiffness mismatch	Pro-inflammatory activation	NF-κB	Fibrous encapsulation
Nanotopography	Cytoskeletal organization	Integrin/FAK	Improved osseointegration

## 2 Biomaterials and immune responses in craniofacial applications

This section examines how physicochemical properties of biomaterials act as upstream regulators of immune activation. Biomaterials used in craniofacial reconstruction not only provide structural support but also actively modulate the host immune response, which critically determines implant integration and bone regeneration outcomes. Beyond their structural role, these materials actively influence the biological microenvironment at the implantation site through physicochemical interactions with proteins, cells, and extracellular matrices. A comparative overview of commonly used craniofacial biomaterials is provided in **Table 2**, emphasizing the relationship between material properties and their immunological profiles. Notably, differences in degradation behavior, surface chemistry, and mechanical properties translate into distinct immune responses that influence implant integration and bone regeneration.

**Table 2:** Comparison of craniofacial biomaterials with emphasis on physicochemical properties and associated immune mechanisms.

Biomaterial	Typical applications	Key physicochemical features	Advantages	Limitations	Immune response and dominant immunological pathways
Titanium alloys	Bone fixation plates, screws	Stable TiO_2_ surface layer; high stiffness; tunable surface roughness	High strength; excellent osseointegration	Particle release possible	Macrophage activation via phagocytosis of wear debris; induction of pro-inflammatory cytokines (e.g., TNF-α, IL-1β, IL-6); activation of NF-κB signaling; modulation of osteoclastogenesis through RANK/RANKL pathway
Stainless steel	Temporary fixation devices	Metallic surface; lower corrosion resistance than titanium	Low cost; high mechanical strength	Corrosion susceptibility; ion release	Release of metal ions induces inflammatory responses; activation of innate immune cells and oxidative stress pathways; increased pro-inflammatory cytokine production
PLA/PLGA	Resorbable fixation	Hydrolytic degradation; controllable degradation kinetics	Biodegradable; eliminates need for removal surgery	Lower mechanical strength; acidic degradation products	Degradation-induced pH decrease alters local immune microenvironment; promotes macrophage activation and inflammatory cytokine release; may sustain pro-inflammatory conditions depending on degradation rate
PCL	Tissue engineering scaffolds	Slow degradation rate; flexible polymer structure	Long-term stability; good processability	Low stiffness; limited load-bearing capacity	Generally mild immune activation; supports macrophage phenotypes associated with tissue repair; may favor pro-regenerative immune responses under controlled conditions
Hydroxyapatite	Bone graft substitute	Calcium phosphate composition similar to bone mineral; high surface bioactivity	Osteoconductive; supports cell adhesion	Brittle; low mechanical strength	Promotes osteoblast activity and supports pro-regenerative immune microenvironment; modulates macrophage polarization toward healing-associated phenotypes
β-TCP	Bone defect filler	Resorbable calcium phosphate; higher solubility than HA	Bioactive; resorbable	Weak mechanical properties	Controlled resorption associated with macrophage-mediated remodeling; supports balanced inflammatory response and bone regeneration
Bioactive glass	Regenerative scaffolds	Ion-releasing surface (e.g., Ca^2+^, Si^4+^); high surface reactivity	Stimulates osteogenesis; bioactive	Brittle; limited mechanical strength	Ionic dissolution products modulate macrophage polarization toward pro-regenerative phenotypes; enhance osteogenic signaling and angiogenesis-related pathways

Metallic biomaterials, particularly titanium and its alloys, remain the most widely used materials in craniofacial fixation systems due to their high mechanical strength, corrosion resistance, and favorable osseointegration properties. Their biocompatibility is largely attributed to the formation of a stable titanium oxide layer, which enhances corrosion resistance and supports bone integration. However, titanium-based materials are not biologically inert, and their interaction with the immune system may be significantly influenced by surface characteristics and the release of wear particles (11). These materials strongly interact with innate immune cells, particularly macrophages, which respond to surface characteristics and wear debris through activation of inflammatory signaling pathways.

Biodegradable polymers such as polylactic acid (PLA), polyglycolic acid (PGA), and their copolymers (PLGA) have been developed as alternatives to permanent metallic implants. These materials undergo hydrolytic degradation *in vivo*, eliminating the need for implant removal. Nevertheless, their degradation products may alter the local pH and modulate inflammatory responses depending on degradation kinetics and concentration (12, 13). Their degradation products can alter the local immune microenvironment, influencing macrophage activation and cytokine production.

Ceramic biomaterials, including hydroxyapatite and β-tricalcium phosphate, are widely used as bone graft substitutes due to their osteoconductive properties and compositional similarity to native bone mineral. These materials support cell adhesion and bone ingrowth, while also influencing immune cell behavior through ionic exchange and surface reactivity. Bioactive glasses further extend this concept by actively releasing ions that stimulate osteogenesis and may promote pro-regenerative immune responses (14). These materials often promote immune environments associated with pro-regenerative macrophage phenotypes.

Composite biomaterials combining polymers and ceramics have attracted increasing attention due to their ability to integrate mechanical stability with biological functionality. Such systems can be engineered to mimic the hierarchical structure of bone, thereby influencing both mechanical performance and cellular responses (15).

Importantly, the biological performance of craniofacial biomaterials is strongly governed by their surface physicochemistry and mechanical properties. Parameters such as surface roughness, wettability, surface energy, and elastic modulus directly regulate protein adsorption, integrin-mediated cell adhesion, and subsequent immune cell activation. For example, moderately rough titanium surfaces have been shown to enhance osteoblast attachment while modulating macrophage polarization toward pro-regenerative phenotypes (16, 17).

Advanced surface engineering techniques, including anodization, sandblasting with acid etching, and plasma spraying, enable precise control of micro- and nanoscale topography. These modifications influence cytoskeletal organization and intracellular signaling pathways, including focal adhesion kinase (FAK) and downstream inflammatory cascades (18). In addition, additive manufacturing technologies allow the fabrication of patient-specific implants with controlled porosity and stiffness, further affecting mechanotransduction and immune cell distribution within the scaffold (19, 20).

Biomaterials physicochemical and mechanical properties define the initial conditions of the biomaterial-tissue interface, thereby shaping macrophage behavior, cytokine signaling, and ultimately bone regeneration outcomes. These physicochemical parameters act as upstream regulators of immune signaling pathways, linking material design with macrophage polarization and downstream regenerative outcomes.

## 3 Titanium particle release and wear debris

Among biomaterials, titanium serves as a key model system to illustrate how material-derived cues initiate immune responses. Titanium-derived wear particles represent a potent trigger of innate immune activation at the biomaterial-tissue interface, playing a central role in implant-associated inflammation and bone remodeling. Titanium and its alloys are widely regarded as the gold standard for craniofacial fixation systems due to their high mechanical strength, corrosion resistance, and favorable biocompatibility, largely attributed to the formation of a stable oxide layer that supports osseointegration (21). However, despite these advantages, titanium-based materials are not biologically inert, and their interaction with the host immune system is strongly influenced by the generation of wear debris.

The biological effects of titanium particles depend on several key parameters, including (i) particle size, (ii) concentration (dose), (iii) physicochemical characteristics, and (iv) the distinction between particulate and ionic forms. These factors critically influence cellular uptake, intracellular signaling activation, and the magnitude of inflammatory responses.

Titanium particles may be generated through several mechanisms, including mechanical wear, fretting at implant interfaces, and electrochemical corrosion processes. Mechanical stresses acting on fixation devices during mastication, facial movements, or postoperative loading can produce microscopic and nanoscopic particles that accumulate in peri-implant tissues. In addition, micromotion between implant components, such as plates and screws, may contribute to the generation of wear debris over time. The biological response to titanium particles is strongly influenced by their physicochemical characteristics, including particle size, morphology, surface charge, and concentration. Nanometer-scale particles are generally more biologically reactive than larger microparticles due to their increased surface area and enhanced cellular uptake (22–24). Macrophages readily internalize titanium particles through phagocytosis or endocytic pathways, which can activate intracellular signaling cascades associated with inflammatory responses. Importantly, titanium ions and particulate forms may induce distinct biological responses, with ionic species primarily affecting signaling pathways through chemical interactions, whereas particles trigger phagocytic uptake and inflammasome activation (25–27). Following particle internalization, macrophages may undergo activation characterized by increased production of pro-inflammatory mediators, including TNF-α, interleukin-1β (IL-1β), and interleukin-6 (IL-6). These cytokines play a central role in regulating bone metabolism by influencing both osteoblast and osteoclast activity. Elevated levels of inflammatory cytokines may promote osteoclast differentiation through activation of the receptor activator of nuclear factor κB (RANK) and receptor activator of nuclear factor κB ligand (RANKL) signaling pathway, thereby contributing to bone resorption in peri-implant regions (28). In addition to macrophage activation, titanium particles may affect other cell types present in the bone microenvironment. Osteoblasts exposed to titanium debris have been reported to exhibit altered proliferation, reduced differentiation, and impaired extracellular matrix production. Endothelial cells may also respond to inflammatory cytokines released by macrophages, which can influence angiogenesis and vascular remodeling during bone healing (29, 30). In addition to classical inflammatory signaling pathways, increasing evidence indicates that titanium particles may activate inflammasome-dependent mechanisms in macrophages. In particular, the NOD-like receptor family pyrin domain containing 3 (NLRP3) inflammasome has been identified as a key intracellular sensor of particulate matter, including metallic debris. Subsequent lysosomal destabilization and reactive oxygen species (ROS) generation may trigger NLRP3 activation, leading to caspase-1 activation and subsequent maturation of IL-1β and IL-18. This pathway represents a critical link between biomaterial-derived stimuli and amplification of inflammatory responses. Clinically, NLRP3 inflammasome activation has been associated with peri-implant inflammation and may contribute to the pathogenesis of peri-implantitis. Importantly, excessive or sustained inflammasome activation may contribute to chronic inflammation and pathological bone resorption in peri-implant tissues, highlighting its relevance as a potential therapeutic target in biomaterial-associated complications.

The accumulation of titanium particles in peri-implant tissues has been documented in both experimental studies and clinical observations. Histological analyses frequently reveal macrophage infiltration and the presence of metallic debris in tissues surrounding craniofacial implants. In some cases, persistent inflammatory responses may lead to fibrous tissue formation and compromised implant integration (31–33).

Recent studies suggest that the immune response to titanium particles represents an important component of osteoimmunological regulation in the peri-implant environment (34). The biological effects of titanium particles are strongly dose-dependent. Low concentrations may support transient immune activation required for tissue repair, whereas high or persistent exposure promotes sustained inflammation and pathological bone resorption.

**Figure 2:**
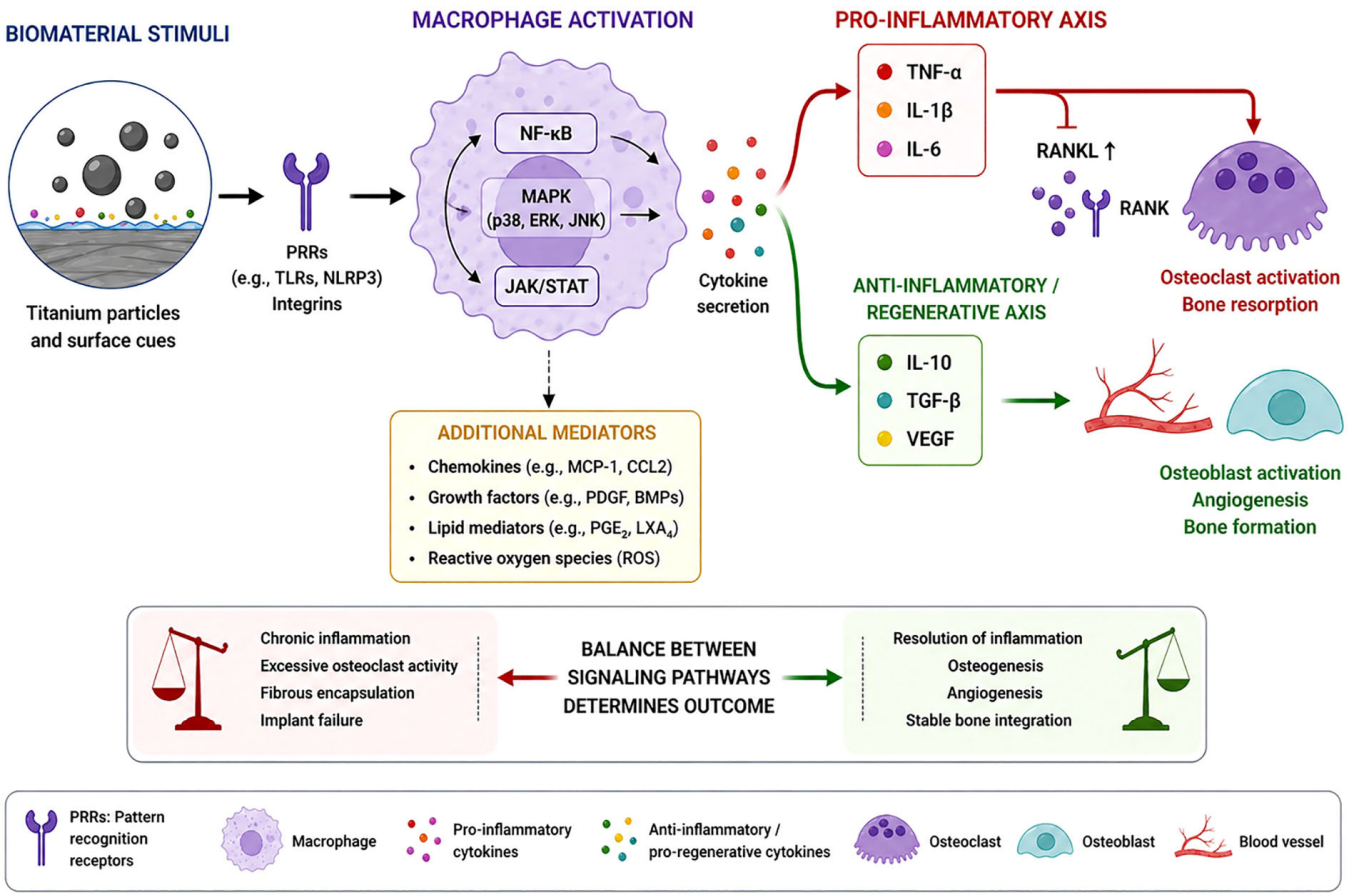
Mechanistic pathways linking biomaterial-derived stimuli with immune activation and bone remodeling. Biomaterial properties, including surface roughness, stiffness, and chemical composition, influence protein adsorption and integrin-mediated signaling pathways. Titanium wear particles activate macrophages and induce polarization toward pro-inflammatory or pro-regenerative phenotypes through NF-κB, MAPK, and STAT6 signaling. These processes regulate cytokine production (e.g., TNF-α, IL-6, IL-10, TGF-β), which in turn modulates osteoclast and osteoblast activity via the RANK/RANKL/OPG axis, ultimately determining bone resorption or regeneration outcomes. (Created in BioRender. (2026) https://BioRender.com/ma1zyy1).

A schematic overview of these processes, integrating titanium particle release with macrophage activation, cytokine signaling, and bone remodeling, is presented in **Figure 2**. This framework highlights how biomaterial-derived cues initiate immune responses that propagate through interconnected signaling pathways to influence osteogenic and osteoclastic activity. From an engineering perspective, strategies aimed at minimizing particle release include optimization of implant surface properties, improvement of mechanical stability, and the development of materials with enhanced wear resistance. In addition, surface modifications that modulate macrophage responses and attenuate pro-inflammatory signaling represent promising approaches to mitigate the adverse effects associated with metallic wear debris and promote regenerative outcomes. These findings establish titanium wear particles as a critical mechanistic link between biomaterial properties, immune activation, and pathological bone remodeling in peri-implant tissues.

## 4 Macrophage polarization in osteoimmunology

At the cellular level, macrophages represent the primary integrators of biomaterial-derived signals. Macrophages are central regulators of the innate immune response to biomaterials, integrating physicochemical cues with intracellular signaling pathways that determine the balance between inflammation and regeneration. As one of the earliest immune cell populations recruited to the implantation site, they orchestrate interactions between immune and stromal cells and play a key role in directing tissue remodeling processes. Importantly, macrophage activation should not be considered a fixed or binary process, but rather a dynamic and context-dependent continuum of functional states shaped by local microenvironmental signals, including those derived from biomaterial surfaces. Macrophages exhibit high phenotypic plasticity and exist along a dynamic spectrum of activation states rather than a strict M1/M2 dichotomy. Their functional phenotype is shaped by microenvironmental cues, including biomaterial-derived signals, cytokines, and metabolic conditions. Pro-inflammatory states are typically associated with NF-κB and MAPK signaling and glycolytic metabolism, whereas pro-regenerative phenotypes involve STAT6 and PI3K/Akt pathways and oxidative metabolism.

Within the simplified M1/M2 framework, pro-inflammatory macrophages are induced by stimuli such as interferon-γ (IFN-γ), microbial products, or damage-associated molecular patterns, and produce cytokines including tumor necrosis factor-α (TNF-α), interleukin-1β (IL-1β), and interleukin-6 (IL-6). These cells are commonly characterized by the expression of markers such as CD86, CD80, and inducible nitric oxide synthase (iNOS) (36–38). In contrast, pro-regenerative macrophages are stimulated by cytokines such as interleukin-4 (IL-4) and interleukin-13 (IL-13) and produce anti-inflammatory mediators including interleukin-10 (IL-10), transforming growth factor-β (TGF-β), and vascular endothelial growth factor (VEGF), supporting angiogenesis, extracellular matrix deposition, and tissue repair. These cells frequently express markers such as CD206 and arginase-1 (Arg-1) and promote osteoblast activity and bone formation (39, 40).

At the molecular level, macrophage polarization is regulated by intracellular signaling pathways activated upon interaction with biomaterial surfaces. Pro-inflammatory polarization is primarily associated with activation of NF-κB and MAPK pathways, whereas pro-regenerative responses are linked to STAT6 and PI3K/Akt signaling following stimulation by IL-4 and IL-13 (41). Biomaterial surface properties modulate these signaling cascades through integrin-mediated adhesion and focal adhesion kinase (FAK) activation, thereby influencing downstream gene expression and cytokine production (42).

This perspective challenges the traditional M1/M2 classification and highlights the context-dependent nature of macrophage activation. Macrophages can adopt intermediate or mixed phenotypes depending on the local microenvironment, including physicochemical cues from biomaterials, soluble mediators, and interactions with surrounding cells (43, 44). In the context of biomaterial implantation, macrophage polarization plays a decisive role in determining the outcome of the host response. An initial pro-inflammatory phase is required for debris clearance and immune activation; however, successful tissue regeneration depends on a timely transition toward pro-regenerative phenotypes that support angiogenesis and matrix remodeling. Persistent pro-inflammatory activation leads to chronic inflammation, fibrous encapsulation, and impaired implant integration (45).

The physicochemical properties of biomaterials are key regulators of macrophage behavior. Surface topography, roughness, wettability, stiffness, and chemical composition regulate macrophage adhesion, morphology, and cytokine production. Nanostructured surfaces that mimic the extracellular matrix promote pro-regenerative macrophage phenotypes, while bioactive modifications and ion-releasing materials modulate intracellular signaling pathways and shift immune responses toward regeneration (46). Macrophages also interact closely with other cell populations involved in bone regeneration. Through the secretion of cytokines and growth factors, they regulate the recruitment and differentiation of osteoblasts, osteoclasts, endothelial cells, and mesenchymal stem cells. This interplay forms the basis of osteoimmunology, bridging immune activation with osteogenesis, angiogenesis, and extracellular matrix remodeling (47).

Consequently, modulation of macrophage polarization through biomaterial design has emerged as a key principle in biomaterials engineering. Biomaterials that promote a controlled transition from pro-inflammatory to pro-regenerative macrophage responses show improved osseointegration and enhanced tissue regeneration (48). A comparative overview of macrophage phenotypes in biomaterial-associated environments is provided in **Table 3**, where the M1/M2 framework is presented as a simplified representation of a broader and dynamic activation spectrum.

**Table 3:** Spectrum of macrophage activation states in biomaterial-associated immune responses.

Characteristic	Pro-inflammatory activation states	Pro-regenerative activation states	Continuum/context-dependence
Key signaling pathways	NF-κB, MAPK, STAT1	STAT6, PI3K/Akt	Significant pathway cross-talk and context-dependent co-activation observed *in vivo*
Activation signals	IFN-γ, microbial stimuli, damage-associated signals	IL-4, IL-13	Overlapping stimuli generate mixed activation states
Cytokines produced	TNF-α, IL-1β, IL-6	IL-10, TGF-β, VEGF	Co-expression of pro- and anti-inflammatory mediators observed *in vivo*
Biological role	Inflammation, pathogen defense, debris clearance	Tissue repair, angiogenesis, extracellular matrix formation	Functions depend on temporal phase of healing and microenvironment
Interaction with biomaterials	Activated by wear particles and pro-inflammatory surface cues; engagement of pattern-recognition pathways and integrin-mediated signaling leading to inflammatory responses	Promoted by bioactive surfaces, ion release, and favorable topographical cues; activation of regenerative signaling pathways supporting tissue integration	Strongly influenced by surface properties and local biochemical cues
Impact on implants	Persistent inflammation, fibrous encapsulation, impaired integration	Enhanced osseointegration and bone regeneration	Outcome depends on balance and timing of activation states
Functional outcome in regeneration	Impaired healing, chronic inflammation	Enhanced regeneration, tissue integration	Transitional phenotypes contribute to both inflammation and regeneration

## 5 Cytokine signaling in implant-associated inflammation

At the molecular level, cytokine signaling networks mediate the downstream effects of macrophage activation. The release of wear particles from biomaterials, particularly titanium-based fixation systems, represents an important trigger of inflammatory signaling at the implant–tissue interface. As discussed in the previous section, titanium particles generated through mechanical wear or corrosion can be internalized by macrophages and other immune cells. This interaction initiates a cascade of cytokine-mediated signaling events that regulate inflammatory responses, cellular recruitment, and bone remodeling in peri-implant tissues.

Cytokines coordinate immune cell communication through tightly controlled signaling pathways in the peri-implant microenvironment. Among these mediators, TNF-α is a central regulator of implant-associated inflammation. TNF-α is primarily produced by activated macrophages following exposure to biomaterial surfaces or wear debris. Binding of TNF-α to its receptors activates intracellular signaling cascades, including the NF-κB, MAPK, and Janus kinase/signal transducer and activator of transcription (JAK/STAT) pathways. These pathways drive transcriptional programs that regulate inflammatory gene expression and amplify immune responses (49–51).

**Figure 3:**
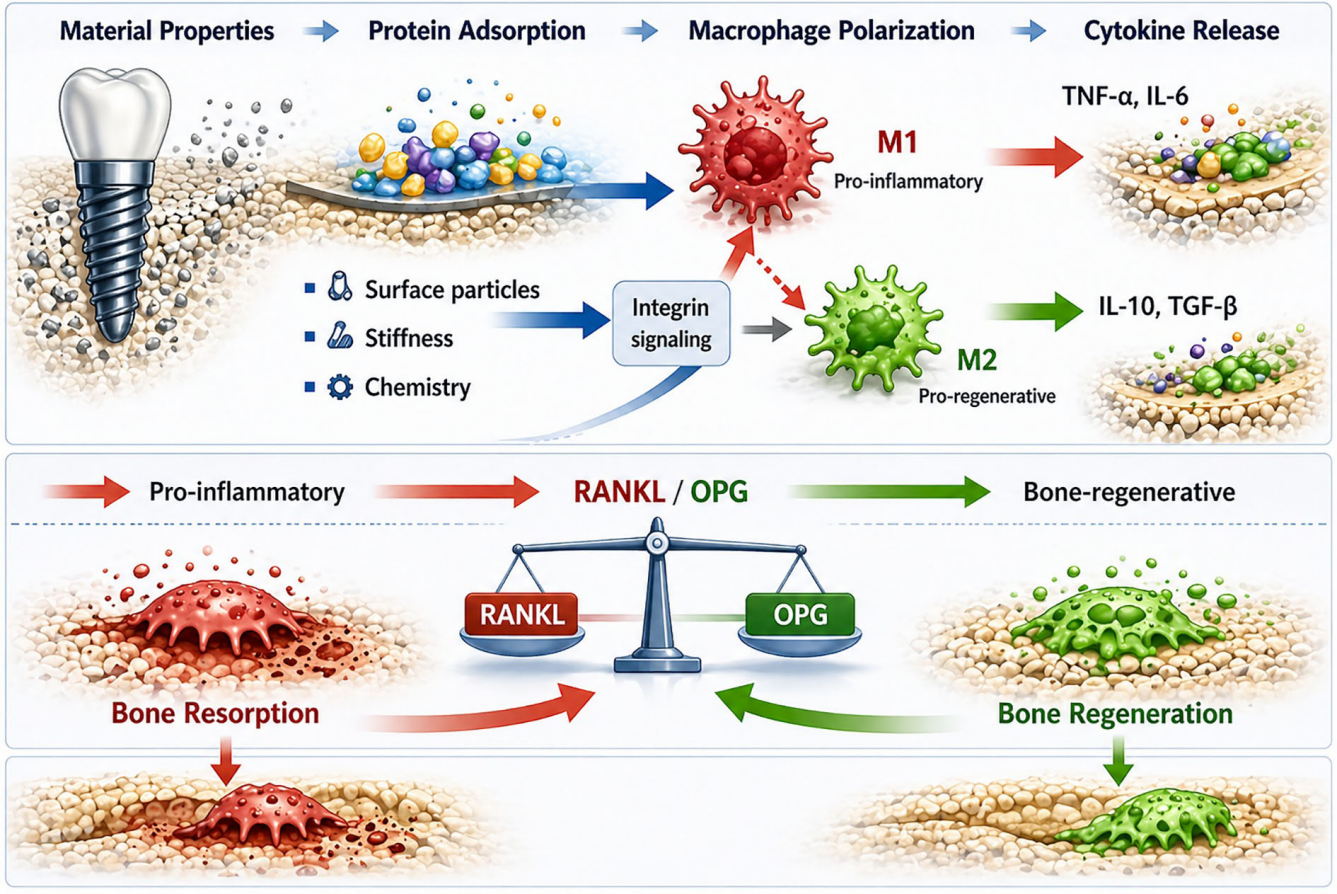
Cytokine signaling network governing biomaterial-induced immune responses and bone remodeling. Macrophage activation by biomaterial-derived stimuli, particularly titanium particles, triggers intracellular signaling pathways including NF-κB, MAPK, and JAK/STAT. These pathways regulate the production of pro-inflammatory cytokines (TNF-α, IL-1β, IL-6), which promote osteoclastogenesis via the RANK/RANKL axis, and anti-inflammatory mediators (IL-10, TGF-β, VEGF), which support osteogenesis and angiogenesis. The balance between these pathways determines regenerative outcomes (Created in BioRender (2026) https://BioRender.com/ydw8i8d).

In addition to TNF-α, several other cytokines participate in the regulation of immune responses surrounding implanted biomaterials. A summary of key cytokines and molecular mediators involved in biomaterial-associated inflammation and bone remodeling is provided in **Table 4**. A schematic overview of the cytokine signaling network and its functional consequences is presented in **Figure 3**. This schematic illustrates how macrophage activation through NF-κB, MAPK, and JAK/STAT pathways leads to the production of pro-inflammatory cytokines (TNF-α, IL-1β, IL-6), promoting osteoclastogenesis via the RANK/RANKL axis. In parallel, anti-inflammatory cytokines (IL-10, TGF-β) and pro-regenerative mediators (VEGF) support osteogenesis and angiogenesis.

**Table 4:** Key cytokines and signaling pathways in biomaterial-associated inflammation and bone remodeling.

Marker	Biological function	Key signaling pathways	Role in biomaterial-associated responses
TNF-α	Pro-inflammatory cytokine	NF-κB, MAPK	Induced by macrophages in response to biomaterial surfaces and wear debris; promotes inflammatory signaling and osteoclastogenesis via RANKL pathway
IL-1β	Pro-inflammatory cytokine	NF-κB, inflammasome pathways	Amplifies inflammatory signaling and enhances osteoclast differentiation through RANKL pathway activation
IL-6	Inflammatory mediator	JAK/STAT3	Regulates immune cell recruitment and contributes to inflammation-mediated bone remodeling
IL-10	Anti-inflammatory cytokine	JAK/STAT3	Suppresses excessive inflammatory responses and promotes resolution of inflammation
TGF-β	Growth factor, pro-regenerative	SMAD signaling	Supports tissue regeneration, extracellular matrix production, and osteogenic differentiation
Caspase-3	Apoptosis effector enzyme	Caspase cascade	Mediates programmed cell death during tissue remodeling; may be activated under inflammatory conditions induced by biomaterials

Pro-inflammatory cytokines such as interleukin-1β (IL-1β) and interleukin-6 (IL-6) amplify inflammatory signaling and promote the recruitment of additional immune cells to the implant site. Conversely, anti-inflammatory mediators including interleukin-10 (IL-10) and transforming growth factor-β (TGF-β) contribute to the resolution of inflammation and the initiation of tissue repair processes. The balance between pro-inflammatory and anti-inflammatory cytokines is therefore a critical determinant of whether the local microenvironment supports regenerative healing or progresses toward chronic inflammation (52).

Cytokine signaling also plays a fundamental role in regulating bone metabolism in the peri-implant environment. Inflammatory mediators influence the activity of both osteoblasts and osteoclasts through several molecular pathways (53). One of the most important regulatory systems is the receptor activator of nuclear factor-κB (RANK), its ligand RANKL, and the decoy receptor osteoprotegerin (OPG). These signaling pathways directly link immune activation with bone remodeling by regulating osteoblast and osteoclast activity. Elevated levels of inflammatory cytokines such as TNF-α and IL-1β can increase RANKL expression, thereby promoting osteoclast differentiation and bone resorption. In contrast, OPG acts as a competitive inhibitor of RANKL and helps maintain bone homeostasis by limiting osteoclast activation (54). Beyond immune cells, cytokine signaling also affects other cell populations involved in bone regeneration. Osteoblasts exposed to inflammatory cytokines may exhibit altered proliferation and differentiation, while endothelial cells respond to cytokine gradients by initiating angiogenic processes necessary for tissue repair. Mesenchymal stem cells recruited to the implant site are similarly influenced by cytokine-mediated signals that regulate their migration and differentiation into osteogenic lineages (55).

The magnitude and duration of cytokine signaling are strongly influenced by the physicochemical properties of biomaterials. These material-dependent cues regulate macrophage activation and intracellular signaling pathways, thereby shaping the cytokine profile at the implant–tissue interface. Surface roughness, chemical composition, and the presence of wear particles can significantly alter macrophage activation and cytokine production. Biomaterials with unfavorable surface characteristics may sustain prolonged inflammatory signaling, whereas materials designed to modulate immune responses can promote a balanced cytokine profile that favors tissue regeneration (56). Collectively, cytokine-mediated signaling networks represent a critical component of the host response to implanted biomaterials. While transient inflammatory signaling is essential for initiating tissue repair, excessive or prolonged cytokine production may disrupt bone regeneration and impair implant integration (57). Consequently, cytokine signaling represents a central mechanistic link between biomaterial properties, innate immune activation, and bone regeneration outcomes (58).

Cytokine signaling in the peri-implant environment should not be considered as a set of isolated pathways but rather as an interconnected network characterized by feedback loops and temporal dynamics. Pro-inflammatory and anti-inflammatory cytokines are co-regulated and often act in a context-dependent manner, where the timing, concentration, and spatial distribution of signals determine biological outcomes. For example, transient TNF-α signaling may support early stages of tissue repair, whereas sustained activation promotes osteoclastogenesis and bone resorption. Collectively, cytokine signaling networks represent a central mechanistic interface linking biomaterial properties, macrophage activation, and bone regeneration outcomes.

## 6 Apoptosis and caspase-3 in tissue remodeling

Apoptosis regulates tissue homeostasis and regeneration by controlling cellular turnover. In the context of biomaterial implantation, apoptotic processes are closely linked to immune activation and inflammatory signaling within the peri-implant microenvironment. These processes are particularly important during bone healing, where the coordinated regulation of cell survival and apoptosis influences the balance between tissue regeneration and degradation (59). Apoptosis is mediated through highly regulated intracellular signaling pathways that can be classified into intrinsic and extrinsic pathways. The intrinsic pathway is typically activated by intracellular stress signals such as oxidative stress, mitochondrial dysfunction, or DNA damage (59). In contrast, the extrinsic pathway is triggered by the activation of death receptors on the cell surface, often in response to inflammatory cytokines including tumor necrosis factor-α (TNF-α). Both pathways ultimately converge on the activation of a family of proteolytic enzymes known as caspases (60). In the context of biomaterial implantation, apoptotic pathways are closely linked to inflammatory signaling induced by material-derived stimuli, including wear particles and cytokine-mediated stress responses.

Caspase-3 is a central executioner of apoptosis. Once activated, caspase-3 cleaves multiple intracellular substrates, leading to characteristic morphological and biochemical changes such as chromatin condensation, membrane blebbing, and DNA fragmentation. Due to its central role in programmed cell death, caspase-3 is widely used as a molecular marker for apoptosis in studies investigating biomaterial–tissue interactions (61). In peri-implant environments, apoptotic signaling can be influenced by inflammatory mediators generated during immune responses to biomaterials. As discussed in the previous section, cytokines such as TNF-α and interleukin-1β may activate signaling pathways that regulate both inflammation and apoptosis. Moderate activation of apoptotic mechanisms contributes to the removal of damaged cells and supports tissue remodeling during the healing process. However, excessive or prolonged activation of apoptosis may impair regeneration by reducing the survival of osteoblasts and other cells involved in bone formation (62, 63).

Biomaterial-related factors may also influence apoptotic processes. The release of wear particles, alterations in local mechanical stress, and oxidative stress associated with inflammatory responses can affect cellular viability in peri-implant tissues. Macrophages and other immune cells exposed to biomaterial debris may release reactive oxygen species and inflammatory mediators that further modulate apoptotic signaling pathways (64). Apoptosis is also closely linked to the regulation of bone remodeling. Controlled apoptotic processes contribute to the removal of osteoclasts after bone resorption and regulate osteoblast turnover during bone formation. Disruption of this balance may lead to impaired osseointegration or excessive bone resorption around implants (65).

Understanding the role of apoptosis and caspase-3 activation in biomaterial-associated tissue remodeling is therefore important for improving implant performance. Biomaterials capable of minimizing excessive inflammatory signaling while maintaining balanced cellular turnover may promote more favorable regenerative outcomes in craniofacial reconstruction. Thus, apoptosis represents a critical regulatory layer linking immune activation with tissue remodeling, and its precise modulation may be essential for optimizing regenerative outcomes in biomaterial-based therapies.

## 7 Immunomodulatory biomaterial design

Immunomodulatory biomaterials represent a paradigm shift from passive biocompatibility toward actively instructive materials capable of directing host immune responses. In this context, immunomodulation can be understood as a controllable design parameter through which biomaterials regulate innate immune responses and downstream regenerative processes. The growing recognition of immune responses as a central determinant of biomaterial performance has led to the emergence of immunomodulatory biomaterials. Unlike traditional biomaterials that were primarily designed to be biologically inert, modern biomaterial strategies aim to actively regulate immune responses in order to promote tissue regeneration and improve implant integration. In particular, the ability to influence macrophage polarization and cytokine signaling has become a key design principle in modern biomaterials engineering. Biomaterials are designed to actively interact with immune cells through controlled physicochemical cues. Surface nano-patterning, for example, can regulate protein adsorption and integrin clustering, thereby influencing macrophage adhesion and polarization. Similarly, the incorporation of bioactive coatings or ion-releasing phases enables spatial and temporal control over local biochemical signaling (67). Engineering approaches also include the use of gradient structures and hierarchical architectures that mimic native bone tissue. These designs influence cell infiltration, vascularization, and immune cell distribution within the scaffold. By integrating structural and biochemical signals, such biomaterials can precisely modulate the immune microenvironment and promote regenerative outcomes (27, 68).

From an engineering perspective, the immunomodulatory performance of biomaterials can be directly linked to quantifiable physicochemical parameters. Moderately rough surfaces often promote more favorable regenerative responses. Similarly, surface wettability, commonly assessed via water contact angle measurements, regulates protein adsorption patterns that subsequently affect integrin-mediated signaling in immune cells (69). Mechanical properties, particularly elastic modulus and stiffness, play a crucial role in mechanotransduction pathways that influence macrophage behavior and osteogenic differentiation. Biomaterials with stiffness values closer to native bone tissue (in the range of several gigapascals for cortical bone) tend to support more balanced immune responses and improved osseointegration (70). In contrast, significant mechanical mismatch may contribute to chronic inflammation or fibrous encapsulation. In addition, nanoscale surface features, including nanotopography and patterning, have been shown to regulate cytoskeletal organization and focal adhesion formation in macrophages, thereby influencing intracellular signaling pathways such as NF-κB and PI3K/Akt (71). The incorporation of controlled ion release systems (e.g., Ca^2+^, Mg^2+^, Si^4+^) further enables temporal modulation of the immune microenvironment, linking material degradation kinetics with biological responses (72). Taken together, these parameters illustrate that immunomodulation is not an abstract concept but a design variable that can be systematically engineered through precise control of material properties. Importantly, these parameters define quantifiable design windows that can be experimentally optimized for specific clinical applications.

One of the most widely explored strategies involves modification of biomaterial surface properties. Surface topography, roughness, and nanoscale architecture can significantly influence macrophage adhesion, morphology, and activation. Nanostructured surfaces that mimic the organization of the natural extracellular matrix have been shown to promote M2-like macrophage phenotypes and reduce excessive inflammatory responses. Similarly, surface wettability and chemical composition can regulate protein adsorption and subsequent immune cell interactions at the biomaterial interface (73). Representative engineering strategies for immunomodulatory biomaterial design are summarized in **Table 5**, highlighting how specific material modifications can regulate immune responses and promote regenerative outcomes.

**Table 5:** Engineering strategies for immunomodulatory biomaterial design and their effects on immune responses.

Strategy	Target immune process	Mechanism of immune modulation and key signaling pathways	Biological effect
Surface nanostructuring	Macrophage polarization	Modulates integrin-mediated adhesion, cytoskeletal organization, and activation of signaling pathways such as NF-κB and PI3K/Akt, influencing macrophage polarization	Promotes pro-regenerative macrophage phenotypes and reduces excessive inflammation
Bioactive coatings	Cytokine signaling	Controlled release of cytokines or bioactive molecules modulates inflammatory signaling pathways and promotes transition toward pro-regenerative macrophage phenotypes	Enhances tissue regeneration and modulates inflammatory responses
Ion-releasing materials	Immune–osteogenic coupling	Ionic signaling (e.g., Ca^2+^, Mg^2+^, Si^4+^) regulates intracellular pathways involved in inflammation and osteogenesis, modulating macrophage polarization and cytokine production	Stimulates osteogenesis and shifts macrophage polarization toward regenerative states
Drug-delivery systems	Inflammation resolution	Enables temporal control of cytokine signaling and inflammatory responses, facilitating the transition from pro-inflammatory to regenerative phases of healing	Reduces chronic inflammation and supports tissue healing

Another important approach involves the incorporation of bioactive molecules into biomaterial surfaces or scaffolds. Cytokines, peptides, and growth factors can be immobilized or released from biomaterials to modulate immune cell behavior and support regenerative processes. For example, biomaterials capable of delivering anti-inflammatory cytokines or pro-regenerative signaling molecules may promote the transition from pro-inflammatory M1 macrophages to regenerative M2 phenotypes, thereby facilitating tissue healing and osseointegration (74).

Ion-releasing biomaterials represent an additional strategy for immunomodulation. Materials capable of releasing biologically active ions such as calcium, magnesium, or silicon can influence both immune and skeletal cells. These ions have been reported to stimulate osteogenic differentiation of mesenchymal stem cells while simultaneously modulating macrophage responses and reducing inflammatory signaling (75).

Drug-delivery systems integrated into biomaterials provide further opportunities to regulate immune responses in a localized and controlled manner. Controlled release of anti-inflammatory agents, immunomodulatory compounds, or growth factors may help create a favorable microenvironment that supports tissue regeneration while preventing excessive inflammatory reactions (76).

Advances in materials engineering and additive manufacturing technologies have also enabled the development of complex scaffold architectures capable of guiding cellular behavior. Three-dimensional printed scaffolds with controlled porosity and microarchitecture can influence immune cell infiltration, vascularization, and tissue integration. By combining structural design with biological functionality, these biomaterials can simultaneously provide mechanical support and actively regulate the host response (77).

Together, these approaches reflect a shift in biomaterials research from passive materials toward immuno-instructive systems capable of actively influencing the healing process. By integrating principles from materials science, immunology, and regenerative medicine, future biomaterials may be designed to precisely regulate immune responses and optimize regenerative outcomes in craniofacial reconstruction (78).

These advances highlight the potential of immunomodulatory biomaterials to actively regulate host responses and improve regenerative outcomes. A comprehensive understanding of the mechanisms discussed in the previous sections is therefore essential for guiding future biomaterial design. Collectively, these approaches demonstrate that immunomodulation can be treated as a tunable design parameter, enabling precise control over the immune microenvironment and regenerative outcomes.

## 8 Discussion

The interaction between biomaterials and the host immune system is increasingly recognized as a central determinant of clinical outcomes in craniofacial surgery (3, 5, 35). Rather than acting as passive structural components, biomaterials actively shape the local immune microenvironment through physicochemical cues that regulate cellular behavior, inflammatory signaling, and tissue remodeling processes (25, 34). This shift from passive biocompatibility toward immune-instructive material design represents a fundamental change in the conceptual framework of biomaterials science (57).

This review highlights that the biological performance of craniofacial biomaterials is governed by a tightly coupled sequence of events linking material properties to immune activation and regenerative outcomes. Material characteristics – including surface topography, chemistry, stiffness, and degradation behavior – define the initial conditions at the biomaterial–tissue interface (17, 18, 46). These parameters regulate protein adsorption and integrin-mediated signaling, which in turn control macrophage activation, cytokine production, and downstream cellular responses such as osteogenesis and angiogenesis (25, 49, 63). This interconnected cascade highlights that biomaterials should be understood as dynamic regulators of immune-mediated regeneration rather than inert scaffolds (35, 56). Taken together, these findings indicate that biomaterial performance cannot be predicted solely based on physicochemical properties, but must be understood as an emergent outcome of dynamic interactions between material-derived cues and the host immune system.

Within this framework, macrophages emerge as central orchestrators of biomaterial-associated responses. Their phenotypic plasticity enables them to integrate physicochemical cues from biomaterials with biochemical signals from the local environment (7, 29, 44). Importantly, macrophage activation does not follow a strict M1/M2 dichotomy but represents a dynamic continuum that evolves during the healing process (43, 44). Successful tissue regeneration requires a coordinated transition from an initial pro-inflammatory phase toward pro-regenerative phenotypes that support angiogenesis, extracellular matrix remodeling, and bone formation (45, 58). Disruption of this transition, for example through persistent exposure to titanium wear debris, may lead to chronic inflammation and impaired implant integration (34). Despite extensive research, the role of macrophage polarization in biomaterial-associated responses remains subject to ongoing debate. While the M1/M2 framework provides a useful conceptual model, increasing evidence suggests that macrophage activation *in vivo* is far more heterogeneous and context-dependent, challenging the predictive value of simplified classifications.

Cytokine signaling networks provide a critical mechanistic link between immune activation and bone remodeling. Rather than acting as isolated mediators, cytokines operate as interconnected and dynamic networks that regulate both the magnitude and temporal progression of immune responses. Pro-inflammatory mediators such as TNF-α and IL-1β promote osteoclastogenesis through activation of the RANK/RANKL pathway, whereas anti-inflammatory cytokines such as IL-10 and TGF-β contribute to the resolution of inflammation and support tissue regeneration (28, 54). These signaling pathways are further modulated by biomaterial properties, which influence both the magnitude and temporal dynamics of cytokine production (52, 56). The balance between pro-inflammatory and pro-regenerative signaling therefore governs osteoclast activity, osteogenesis, and overall regenerative outcomes. Importantly, this balance is not solely biologically determined but can be actively modulated through biomaterial design (56).

In addition to inflammatory signaling, apoptosis represents an important regulatory mechanism in the peri-implant environment. Caspase-3-mediated apoptotic pathways contribute to cellular turnover and tissue remodeling, linking immune activation with the removal of damaged or dysfunctional cells (60). The interplay between cytokine signaling and apoptosis further underscores the complexity of biomaterial–immune interactions and highlights the need for integrated design strategies that consider multiple biological processes simultaneously (62, 63).

The convergence of these mechanisms supports a design paradigm in which immunomodulation is treated as a controllable material parameter. Strategies such as surface nanoengineering, ion release, bioactive coatings, and drug-delivery systems enable the precise regulation of immune responses at the biomaterial interface (27, 66, 73). Rather than suppressing inflammation, these approaches aim to modulate its magnitude and temporal progression in order to promote regenerative outcomes, consistent with evidence that a controlled inflammatory response is essential for effective tissue repair (28, 62).

From a clinical perspective, dysregulated immune responses to biomaterials are directly associated with adverse outcomes such as chronic inflammation, fibrous encapsulation, and peri-implant bone loss (31–34). In craniofacial applications, these processes are particularly relevant in the context of implant instability and peri-implantitis, where persistent inflammatory signaling driven by macrophage activation and cytokine imbalance can impair osseointegration. Understanding the osteoimmunological mechanisms described in this review provides a basis for developing clinically relevant strategies aimed at improving implant performance. Specifically, biomaterials designed to modulate early innate immune responses, promote controlled macrophage transitions, and regulate cytokine dynamics may reduce complication rates and enhance long-term implant stability (31–34).

Despite growing interest in immunomodulatory biomaterials, their clinical translation remains limited. Most current systems remain at low to intermediate technology readiness levels (TRL 2–5), with only a small subset progressing toward preclinical validation or early clinical evaluation. Key barriers to translation include insufficient standardization of *in vitro* and *in vivo* models, limited reproducibility across studies, and the lack of predictive frameworks governing immune responses to long-term clinical outcomes (35). In addition, regulatory pathways for immunomodulatory biomaterials remain complex, as these systems often combine material, biological, and drug-like functionalities, placing them at the interface of medical devices and advanced therapy medicinal products. Addressing these challenges will require the development of standardized testing protocols, improved preclinical models, and closer integration between materials science, immunology, and regulatory science.

Despite significant progress, several challenges remain in translating osteoimmunological principles into clinical applications. One major limitation is the oversimplification of macrophage polarization using the M1/M2 classification, which does not fully capture the heterogeneity of macrophage phenotypes observed *in vivo* (38, 44). Furthermore, many experimental models fail to reproduce the complexity of clinical conditions, where factors such as mechanical loading, vascularization, and systemic immune responses play critical roles (10, 22). A major limitation in the field is the reliance on simplified *in vitro* models that fail to capture the complexity of the *in vivo* immune microenvironment, including dynamic mechanical loading, vascularization, and systemic immune interactions. As a result, biomaterials that demonstrate promising immunomodulatory properties *in vitro* may not achieve comparable performance in clinical settings (35).

Future research should focus on developing predictive models that integrate material properties with immune responses and regenerative outcomes. Advances in systems biology, high-throughput screening, and computational modeling may enable the identification of key design parameters that govern biomaterial performance (44, 53). In addition, the development of standardized experimental frameworks and clinically relevant models will be essential for improving the translational potential of immunomodulatory biomaterials (35, 56).

The findings discussed in this review support the concept that successful craniofacial biomaterials must be designed as active regulators of the immune microenvironment. By integrating materials science with immunology, it becomes possible to develop next-generation implants that not only provide structural support but also guide the healing process toward predictable and regenerative outcomes (48).

Furthermore, the biological impact of titanium wear particles remains controversial, as their effects appear to depend on particle size, concentration, and local microenvironmental conditions. While some studies report predominantly pro-inflammatory effects, others suggest that controlled exposure may also contribute to regenerative signaling, highlighting the complexity of biomaterial-induced immune responses. Therefore, future progress in craniofacial biomaterials will depend on the development of integrative design strategies that explicitly incorporate immunological principles as key determinants of regenerative success.

## 9 Future perspective

The growing integration of immunology and biomaterials science is expected to play a defining role in the next generation of craniofacial reconstruction strategies. Increasing evidence indicates that the success of implanted biomaterials depends not only on their mechanical and structural properties but also on their ability to actively modulate the host immune response (25, 47). Consequently, future biomaterial design will increasingly focus on systems capable of directing immune processes toward regenerative outcomes rather than merely minimizing adverse reactions. A major challenge in the field is the limited ability to precisely control immune responses in a spatially and temporally defined manner within the complex *in vivo* environment.

One of the most promising directions involves the development of immuno-instructive biomaterials that precisely regulate macrophage polarization and cytokine signaling at the implant interface (45, 48). Advances in nanotechnology and surface engineering enable fine control over topography, chemistry, and mechanical properties, which are known to influence immune cell behavior and intracellular signaling pathways (17, 46, 71). In particular, biomimetic and nanostructured surfaces designed to replicate the architecture of the extracellular matrix may promote pro-regenerative immune responses and enhance osseointegration (46, 73).

Another emerging strategy involves the incorporation of controlled delivery systems for bioactive molecules. Biomaterials capable of releasing cytokines, growth factors, or anti-inflammatory agents in a spatially and temporally controlled manner may enable precise regulation of the local immune microenvironment (56, 76). Similarly, ion-releasing materials that deliver biologically active ions such as calcium, magnesium, and silicon provide an additional mechanism for modulating both immune and skeletal cell responses (72, 75). In this context, adaptive and stimuli-responsive biomaterials capable of dynamically interacting with the immune system represent a promising direction for next-generation implant design.

Additive manufacturing technologies, including three-dimensional printing, further expand the design space for craniofacial biomaterials by enabling the fabrication of patient-specific implants with controlled architecture and mechanical properties (20, 77). Such approaches allow the integration of structural and biological functionality, facilitating improved vascularization, immune cell infiltration, and tissue regeneration.

Despite these advances, significant challenges remain in translating immunomodulatory biomaterials from experimental models to clinical practice. Current *in vitro* and *in vivo* models often fail to capture the complexity of human immune responses, particularly in the context of dynamic mechanical environments and patient-specific variability (10, 22). Addressing these limitations will require the development of more predictive experimental systems and standardized evaluation protocols.

Future progress in this field will likely depend on the integration of materials science with systems biology and data-driven approaches. High-throughput screening, omics-based analyses, and computational modeling may enable the identification of key parameters governing biomaterial–immune interactions and facilitate the rational design of personalized biomaterials tailored to individual immune profiles (44, 53). Future approaches may also involve the development of personalized biomaterials tailored to patient-specific immune profiles, enabling more precise and predictable regenerative outcomes.

Ultimately, the convergence of biomaterials engineering, immunology, and regenerative medicine is expected to lead to the development of adaptive, immuno-instructive implants capable of dynamically interacting with the host environment. Such systems may enable more predictable healing outcomes and represent a significant step toward personalized and precision medicine in craniofacial reconstruction. Ultimately, the integration of immunology, materials science, and data-driven approaches is expected to enable the development of predictive and immune-instructive biomaterials capable of guiding tissue regeneration in a controlled and reproducible manner.

Future studies should focus on integrating quantitative design parameters with predictive immunological models to enable rational and clinically translatable biomaterial design. The development of standardized osteoimmunological testing platforms will be essential to bridge the gap between experimental findings and clinical applications.

## 10 Conclusion

Biomaterials used in craniofacial applications should be regarded not as passive structural components, but as active regulators of the immune microenvironment that governs tissue regeneration and implant integration. The interplay between material properties, macrophage-mediated immune responses, cytokine signaling, and apoptosis pathways defines the biological outcome at the biomaterial–tissue interface. These interconnected processes highlight that successful biomaterial design requires integration of physicochemical parameters with immune signaling mechanisms. These findings highlight immune regulation as a primary determinant of biomaterial-driven regeneration.

Macrophages emerge as key orchestrators of these processes, with their dynamic and context-dependent activation states determining the balance between inflammation and regeneration. In this context, factors such as titanium wear debris and biomaterial surface characteristics critically influence immune activation and downstream bone remodeling.

The growing understanding of osteoimmunological mechanisms has enabled the development of immunomodulatory biomaterials designed to actively direct host responses. By integrating physicochemical design parameters with immune signaling pathways, it becomes possible to shift biomaterial performance from passive compatibility toward controlled, regenerative functionality.

Future progress in craniofacial biomaterials will depend on the development of immune-instructive systems capable of precisely modulating host responses to achieve predictable and regenerative healing outcomes.
